# Combined use of baking soda and electric toothbrushing for removal of artificial extrinsic stain on enamel surface: An *in vitro* study

**DOI:** 10.4317/jced.58708

**Published:** 2022-01-01

**Authors:** Akiko Haruyama, Masashi Kojima, Atsushi Kameyama, Takashi Muramatsu

**Affiliations:** 1 Senior Assistant Professor, Department of Operative Dentistry, Cariology and Pulp Biology, Tokyo Dental College, 2-9-18, Kanda-misakicho, Chiyoda-ku, Tokyo 101-0061, Japan; 2 Dental Research Student, Department of Operative Dentistry, Cariology and Pulp Biology, Tokyo Dental College, 2-9-18, Kanda-misakicho, Chiyoda-ku, Tokyo 101-0061, Japan; 3 Professor, Department of Operative Dentistry, Endodontology, and Periodontology, School of Dentistry, Matsumoto Dental University, 1780 Gobara Hirooka, Shiojiri, Nagano 399-0781, Japan; Professor, Department of Oral Health Promotion, Graduate School of Oral Medicine, Matsumoto Dental University, 1780 Gobara Hirooka, Shiojiri, Nagano 399-0781, Japan; 4 Professor and Chairperson, Department of Operative Dentistry, Cariology and Pulp Biology, Tokyo Dental College, 2-9-18, Kanda-misakicho, Chiyoda-ku, Tokyo 101-0061, Japan

## Abstract

**Background:**

This study aimed to investigate the combined effect of baking soda and electric toothbrushing on the removal of artificial extrinsic stain *in vitro*.

**Material and Methods:**

Flat enamel surfaces of 15 bovine incisors were artificially stained with 10% citric acid / 3% ferric chloride solution followed by 1% tannic acid solution. These specimens were randomly divided into three groups (n = 5) – Group S+B: brushing with an electric toothbrush and baking soda, Group S+C: brushing with an electric toothbrush and fluoride dentifrice, Group S: brushing only with an electric toothbrush. Color values (L*, a*, and b*) and surface roughness were measured before and after brushing (after 1, 2, 3, and 5 min). The data were statistically analyzed using two-way analysis of variance and Tukey’s honest significant difference test as a post hoc test (*p*< 0.05).

**Results:**

The L* value of Group S+B increased over time, and was significantly different between before brushing and at 5 min (*p*< 0.05). A significant difference in the ΔE* value of Group S+B was found at 5 min (*p*< 0.05). However, no significant difference was found in the ΔE* values of Group S+C and Group S. No significant differences in Ra were found in any of the groups.

**Conclusions:**

The results of this study suggest that the combined use of baking soda and electric toothbrushing has an excellent stain-removing effect compared with electric toothbrushing with a fluoride dentifrice. Additionally, the changes in surface roughness were similar to the changes caused by the use of general dentifrices.

** Key words:**Baking soda, dentifrice, extrinsic stain removal, color change, surface roughness.

## Introduction

White and healthy teeth are increasingly considered desirable, and many patients request tooth whitening. Deposition of stain on the tooth surface is caused by the adsorption of food and drink pigments on the pellicle on the enamel surface; for example, by the ingestion of coffee, tea, red wine, and spicy foods (1). Tooth discoloration can also be caused by poor brushing technique, smoking, the use of cationic antiseptics such as chlorhexidine and cetylpyridinium chloride, and the intake of metal salts such as iron and tin (2,3). Stained teeth do not look healthy or esthetically pleasing, and professional care or self-care for removing stains is recommended.

Several methods for removing stains have been reported, including professional care by a dentist or dental hygienist/dental nurse or self-care with a toothbrush. Professional care, such as mechanical prophylaxis (4) with polishing paste or air-powder polishing combining water and granular sodium hydrogen carbonate (baking soda) removes staining, but it requires time and effort as well as treatment costs each time. In contrast, self-care using dentifrice is easier than professional care because it is done at home. In particular, self-brushing using an electric/sonic toothbrush has been reported to provide a stain removal effect that is superior to manual brushing with toothpaste (5).

Abrasives are included in dentifrices for removing discoloration on the tooth surface (6,7). In recent years, to improve the whitening effect, dentifrices containing whitening additives, such as sodium polyacrylate, sodium pyrophosphate, and baking soda, have become commercially available (2,8). Baking soda, also used for air-powder polishing, is used as a household detergent, and is biologically compatible with little adverse effect on the environment on account of its acid-buffering capacities, antibacterial properties at high concentrations, and relatively low abrasivity (9,10). The whitening effect of a dentifrice depends largely on the abrasive compounds (7); however, the whitening effect of baking soda combined with electric toothbrushing and the resulting surface changes in the enamel require further elucidation.

Therefore, the purpose of this study was to observe the effect of removing stains and the change in surface roughness of the tooth surface caused by the use of baking soda in combination with an electric toothbrush *in vitro*. The null hypotheses in this study are: 1) that the use of a dentifrice containing baking soda combined with electric toothbrushing has the same effect as a commercially available dentifrice combined with electric toothbrushing, and 2) that the use of a dentifrice containing baking soda combined with electric toothbrushing roughens the tooth surface.

## Material and Methods

-Specimen preparation

Fifteen extracted bovine teeth (Yokohama Meat Corporation, Yokohama, Japan) frozen to maintain freshness, were defrosted and cut at the cervix using a diamond cut saw (KT100, Maruto Instrument Co., Tokyo, Japan). The dental pulp tissue was removed from the crown portions using an #80 K-file (Mani, Tokyo, Japan). The pulp cavity was then filled with auto-cured acrylic resin (Unifast III, GC, Tokyo, Japan), and the crown was embedded in an acrylic ring (Refine Tech, Yokohama, Japan) using epoxy resin (Scandiprex, Fritsch Japan, Yokohama, Japan). The embedded specimens were abraded with #240 SiC paper to obtain a flat enamel surface using an automatic polishing machine (Automet 250, Buehler, IL, USA).

-Artificial staining procedure 

Artificial staining was carried out according to the method of our earlier study (11). In brief, the prepared enamel surfaces were treated with an aqueous solution of 10 wt% citric acid / 3 wt% ferric chloride (10-3 PRE-treating agent, Nippon Shiken Corporation, Tokyo, Japan) for 60 s and then thoroughly rinsed with water spray. The specimens were immersed in chicken egg white for 10 min and then immersed in a 1 wt% aqueous solution of tannic acid (Wako Pure Chemicals, Osaka, Japan). Upon completion of the staining process, the specimens were rinsed with running tap water, and stored in 37°C water for 1 week.

-Brushing protocol

Specimens were randomly divided into the following three groups (n = 5).

Group S+B: Brushing with an electric toothbrush (Sonicare Easy Clean, Phillips Oral Healthcare, Bothell, WA, USA) and Magic Powder Tengai Ten Syringol Baking Soda (Niwakyu, Gifu, Japan) 

Group S+C: Brushing with an electric toothbrush (Sonicare Easy Clean) and Clinica fluoride-containing dentifrice (Lion, Tokyo, Japan) 

Group S: Brushing with an electric toothbrush (Sonicare Easy Clean) with no dentifrice.

Each specimen was fixed onto a disposable dish with double-sided tape and placed on a kitchen scale. A small amount of tap water and a rice-grain-size amount of dentifrice or baking soda was placed on the central portion of the enamel surface and the specimen was brushed with hand pressure to maintain a loading of 90 gf. Brushing was performed for 1 min, 2 min, 3 min, and 5 min for each sample. After the brushing procedure, the specimens were washed with water spray and dried with delicate task wipe towels (KimWipes, Nippon Paper Crecia, Tokyo, Japan).

-Color change measurements

The color of each specimen was measured with a microscopic area spectrophotometer (MSP-300H, Nippon Denshoku Industries, Tokyo, Japan), using the CIE L*a*b* color system, where the L* axis indicates the value (lightness/darkness), the a* axis represents the redness (+a*) or greenness (-a*), and the b* axis demonstrates the yellowness (+b*) or blueness (-b*).

The color of each specimen was measured at 0 min (L*0 min, a*0 min, b*0 min), 1 min (L*1 min, a*1 min, b*1 min), 2 min (L*2 min, a*2 min, b*2 min), 3 min (L*3 min, a*3 min, b*3 min), and after the completion of brushing (L*5 min, a*5 min, b*5 min). Color was measured five times for each specimen, and the average values obtained were defined as the color values of each specimen. Next, the differences between the L*, a*, and b* values at each time point and the values before brushing were calculated as follows:

ΔL* = L*1/2/3/5 min- L*0 min 

Δa* = a*1/2/3/5 min- a*0 min 

Δb* = b*1/2/3/5 min- b*0 min 

The color change (ΔE*) was calculated using the following equation.

ΔE* = [(ΔL*)2 + (Δa*)2 + (Δb*)2]1/2 

The material was photographed with a camera (Nikon D5600, Nikon, Tokyo, Japan) before and after staining, and 5 min after brushing for each group.

-Surface roughness measurement (Ra)

Surface roughness was measured to observe changes in the enamel surface before and after brushing for 1, 2, 3, and 5 min using a surface profilometer (Surfcom 130A, Tokyo Seimitsu, Tokyo, Japan), with a standard cutoff of 0.8 mm, a transverse length of 4.0 mm, and a stylus speed of 0.6 mm/s. By changing angles, measurements were performed at five sites near the center to calculate the mean surface roughness (Ra).

The material was photographed with a 3D measuring laser microscope (LEXT OLS4000, Olympus, Tokyo, Japan) before and after staining and 5 min after brushing in each group.

-Statistical analysis

The obtained data were used to calculate the mean and standard deviation (SD) for each group, and were statistically analyzed using two-way analysis of variance (ANOVA), followed by Tukey’s honestly significant difference (HSD) test with statistical significance set at a *p-value* of 0.05. All statistical analyses were performed using IBM SPSS statistics 18 for Windows (IBM Japan Inc., Tokyo, Japan).

## Results

-Color change measurements

**Figure 1:**
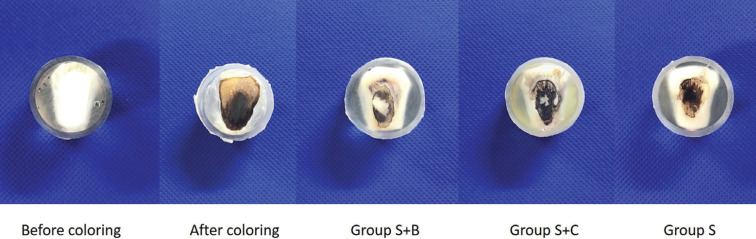
Representative photographs of the enamel surface before staining, after staining, and after treatment (Groups S+B, S+C, and S).

Representative photographs are shown in Figure 1. The staining was removed in Groups S+B and S+C, but not in Group S. The results of the changes in individual color parameters are shown in Table 1, and the color change (ΔE*) results are shown in Table 2. In the L* value indicating the brightness, no change with time were observed in Group S. Groups S+B and S+C showed an increase over time, with a significant difference between before brushing and 5 min after brushing (*p* < 0.05). The a* values in all groups did not change over time after 1 min. In terms of b* values, Group S+B showed an increase over time (*p* < 0.05). In ΔE*, a significant change over time was observed in Group S+B (*p* < 0.05).

**Table 1:**
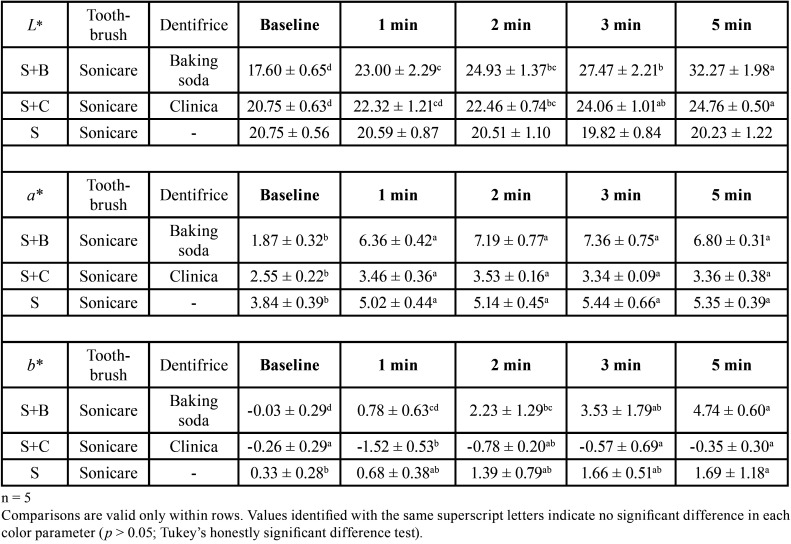
Means and standard deviations for L* values, a* values and b* values.

**Table 2:**
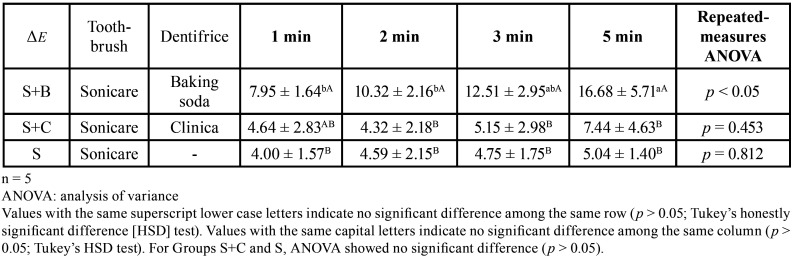
Means and standard deviations for ΔE values.

-Surface roughness measurement (Ra)

**Figure 2:**
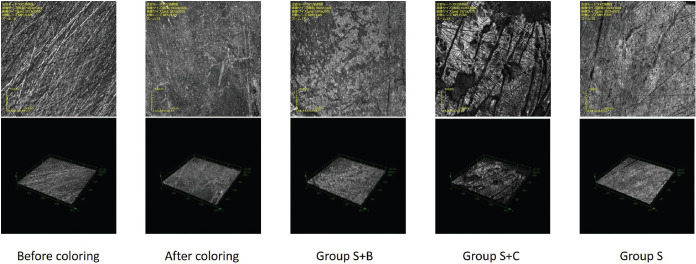
Micrographs taken with the 3D measurement laser microscope of the enamel surface before staining, after staining, and after treatment (Groups S+B, S+C, and S). Upper: Secondary electron image. Lower: 3D display image.

The results of the changes in individual surface roughness are shown in  Table 3. No significant differences were observed in Ra values in any of the groups (*p* > 0.05). The representative photographs of the 3D measurement laser microscope are shown in Figure 2. In the comparison of before and after staining, it can be confirmed that the scratches on the polished surface before staining are not clear after staining, confirming that the coloring was removed in all the groups. The scars were no larger after brushing than before brushing in any of the groups.

**Table 3:**
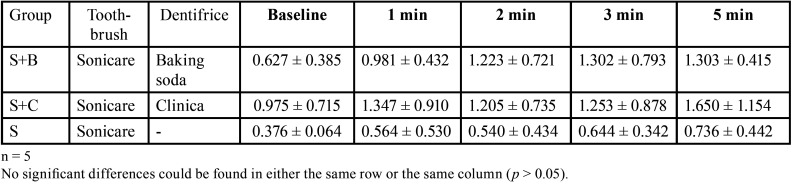
Difference in surface roughness measured before and after prophylaxis (mean ± SD, μm, n = 5).

## Discussion

The purpose of this study was to observe the stain-removing effects and the changes in the tooth surface roughness caused by the use of baking soda in combination with electric toothbrushing *in vitro*. The results of this study revealed that the extrinsic stain removal effect achieved with the combined use of baking soda and electric toothbrushing was more effective than electric toothbrushing with commercially available dentifrices. Therefore, the first null hypothesis (that the use of a dentifrice containing baking soda combined with electric toothbrushing has the same effect as a commercially available dentifrice combined with electric toothbrushing) was rejected.

Researchers have commonly used aqueous solutions such as tea or instant coffee as an artificial staining solution when evaluating the whitening of discolored teeth *in vitro* (12). However, it is desirable to deposit artificial stain only on the surface of the enamel without discoloring the interior of the tooth substrate when evaluating the removal of extrinsic staining. In this study, we employed 10% citric acid / 3% ferric chloride aqueous solution (10-3 solution, as used in the pretreatment for dentin bonding of 4-META/MMA-TBB resin) (13) and tannic acid aqueous solution to generate extrinsic artificial stain for the enamel surface. Fe ions (III) combine with tannic acid to produce brown or black ferric tannate when exposed to air (14). Natural staining of the enamel surface may be formed by a similar mechanism (15). Our previous studies have shown that this method does not discolor the interior of the dentin and can stain only the enamel surface (11). In this study, an extrinsic artificial stain was generated by the same method.

Because the Mohs hardness number of baking soda is approximately 2.5, its polishing effect is thought to be lower than that of silica. However, the increase in L* value between before brushing and after 5 min was greater in Group S+B than in Group S+C, suggesting there are factors other than the hardness of the abrasive. Our results are consistent with the results of previous reports by Yankell *et al*. who showed that baking soda dentifrices are superior to conventional silica-based dentifrices in removing extrinsic stain (16), and by Putt *et al*. who demonstrated *in vivo* (17). The complex of tannic acid and Fe ions is hydrolyzed by adding baking soda in the presence of oxygen to form carboxylic acid, which is further neutralized by the baking soda. These findings suggest that the stain in our study may have decomposed.

The values of a* and b* in Group S and Group S+C, no significant changes over time were observed before and after brushing. In contrast, in Group S+B were significantly higher than in the other groups. These results indicate that the color tone changed in the red and yellow directions. When hydrolyzed by baking soda, tannic acid becomes gallic acid, and the hydroxyl group reacts with Fe2+ on the tooth surface to form a complex (18). At this time, the complex is reddish brown, but it is then oxidized by air to become a black Fe3+ complex. It is thought that the measurements in this study took place immediately after formation of the Fe3+ complex, and that the Fe3+ on the tooth surface reacted with the base (OH-) in the baking soda to produce iron (III) oxide (Fe3++ 3OH- → 3Fe (OH)). Because iron (III) oxide (Fe2O3) is reddish brown, a* and b* values might be increased. Therefore, in Group S+B, large changes in L*, a*, and b* values were reflected in ΔE*, which was significantly different from Group S+C and Group S.

The Clinica dentifrice used in this study is a silica containing dentifrice that enhances the cleaning effect during brushing because Mohs hardness number of silica is approximately 7. In fact, no change in L* value was observed over time in Group S, but an increase was observed in the L* value over time in Group S+C. These results may be due to the exfoliation of the stain on the enamel surface by silica. There was no change in Ra over time for brushing alone without dentifrice (Group S) or for brushing with Clinica dentifrice (Group S+C). These findings support the effect of silica on stain removal. It has been reported that the Sonicare toothbrush caused no significant increase in the surface roughness or the wear on sound dental enamel (19). Arends *et al*. evaluated the amount of enamel wear on the tooth surface when a commercially available dentifrice was used, and they concluded that the dentifrice caused little wear (20). The results of our study also revealed no significant difference in Ra regardless of whether dentifrice was used or not. Additionally, there was no significant difference in Ra values in Groups S+B and S+C. As was the case with silica, baking soda had little effect on the roughness of the enamel surface. Therefore, the second null hypothesis (that the use of a dentifrice containing baking soda combined with electric toothbrushing roughens the tooth surface) was rejected.

Electric toothbrushes such as Sonicare have been reported to remove plaque more effectively than manual toothbrushes (21,22). Dawson *et al*. (14) reported that brushing with a manual toothbrush did not remove much exogenous stains from the surface of ferric tannate-stained hydroxyapatite discs. Previously revealed that the Sonicare toothbrush, when used in combination with dentifrice, effectively removes exogenous artificial stains from the enamel surface. Brushing was performed with a load of 90 gf, as recommended by the manufacturer and previous studies (14,23). Sorensen *et al*. found that increasing the force of the toothbrush from 90gf to 150gf increased the wear of the dentin substrate (24). The Sonicare toothbrush description recommends brushing the entire dentition for 2 minutes each time (25,26). This is equivalent to brushing the surface of each tooth for about 2 seconds. Therefore, brushing specimens for 1, 2, 3, and 5 minutes corresponds to normal brushing for approximately 10, 20, 30, and 50 days, respectively. In conclusion, the 50 days combined use of an electric toothbrush and baking soda showed excellent stain-removing properties when compared with the use of an electric toothbrush and a commercially available dentifrice, without altering the surface roughness of the enamel. Baking soda is known to enhance plaque removal because it reduces the viscosity of plaque polysaccharides and binds to calcium ions to loosen the bonds between bacteria (27). Furthermore, its biological acid buffering capacity and antibacterial effect contribute to the prevention of oral malodor and tongue coating (28). Baking soda should be recommended to consumers not only to help remove dental stains, but also to improve general oral health.
